# Multi-Morbidity in Hospitalised Older Patients: Who Are the Complex Elderly?

**DOI:** 10.1371/journal.pone.0145372

**Published:** 2015-12-30

**Authors:** Milagros Ruiz, Alex Bottle, Susannah Long, Paul Aylin

**Affiliations:** 1 Dr Foster Unit at Imperial, Dept. Primary Care and Public Health, School of Public Health, Imperial College London, Reynolds Building, St. Dunstan's Road, London, W6 6RP, United Kingdom; 2 Department of Medicine for the Elderly, Cambridge Wing, St Mary's Campus, Imperial College London, Praed Street, London, W2 1NY, United Kingdom; University of Naples Federico II, ITALY

## Abstract

**Background:**

No formal definition for the *“complex elderly”* exists; moreover, these older patients with high levels of multi-morbidity are not readily identified as such at point of hospitalisation, thus missing a valuable opportunity to manage the older patient appropriately within the hospital setting.

**Objectives:**

To empirically identify the complex elderly patient based on degree of multi-morbidity.

**Design:**

Retrospective observational study using administrative data.

**Setting:**

English hospitals during the financial year 2012–13.

**Subjects:**

All admitted patients aged 65 years and over.

**Methods:**

By using exploratory analysis (correspondence analysis) we identify multi-morbidity groups based on 20 target conditions whose hospital prevalence was ≥ 1%.

**Results:**

We examined a total of 2788900 hospital admissions. Multi-morbidity was highly prevalent, 62.8% had 2 or more of the targeted conditions while 4.7% had six or more. Multi-morbidity increased with age from 56% (65-69yr age-groups) up to 67% (80-84yr age-group). The average multi-morbidity was 3.2±1.2 (SD). Correspondence analysis revealed 3 distinct groups of older patients. Group 1 (multi-morbidity ≤2), associated with cancer and/or metastasis; Group 2 (multi-morbidity of 3, 4 or 5), associated with chronic pulmonary disease, lung disease, rheumatism and osteoporosis; finally Group 3 with the highest level of multi-morbidity (≥6) and associated with heart failure, cerebrovascular accident, diabetes, hypertension and myocardial infarction.

**Conclusions:**

By using widely available hospital administrative data, we propose patients in Groups 2 and 3 to be identified as the *complex elderly*. Identification of multi-morbidity patterns can help to predict the needs of the older patient and improve resource provision.

## Introduction

Hospital admissions of older patients are in rapid increase and their care management is high in the agenda of clinicians and policy makers **[1]**. Between 2002 and 2012, the number of patients admitted to NHS hospitals in England who were aged 75 and over rose by almost two thirds **[2]**, furthermore, 65% of people admitted to hospitals were older than 65 years and accounted for 70% of bed days **[3]**; as the proportion of people aged 60 and over is increasing at a faster rate than any other age group **[4]**, demand for hospital care by the oldest patient population will continue to rise **[5]**.

Studies have shown that hospitalised older patients will often present mental, psychological and/or social difficulties requiring coordinated care from different health specialities **[3, 6]**. These patients are often referred to as the “*complex elderly*” or the “*older patient with complex needs*” in an attempt to reflect the complexity of care needed by these patients of advanced age with multiple simultaneous medical conditions **[7]**. Although the *complex elderly* label is deemed inappropriate by some (personal communication, British Geriatrics Society, 2012), it is a term widely used in medical research and health literature, but so far there is no consensus on a definition, empirical or otherwise. Past attempts to define the *“complex patient”* have incorporated parameters such as the overall cost of care, the need for multiple interventions (referrals), and/or complexity of clinical profile **[8–10].** In the past, the *complex elderly* term has been used by health organisations to establish charging costs for this particular group of patients who are known to consume considerable hospital financial resources. For example, the Healthcare Resource Group (HRG) algorithm within the English National Health Service (NHS) tries to group patients consuming similar levels of resources (similar to the Diagnosis-Related Group system). In this context, HRG (version 3.5, 2007) identified the *complex elderly* as those patients having two or more major diagnoses, with no significant procedure recorded and whose age was greater than 69 years. It is worth mentioning that the HRG grouping for the *complex elderly* has not been included in their latest version 4 (HRG4, released Feb 2013), but it is under review for future releases.

More recently, patient complexity assessment has progressed towards the inclusion of psychological, socioeconomic, cultural, environmental, behavioural as well as biological factors **[11–13].** For this purpose, the Interdisciplinary Medicine Instrument (INTERMED) tool was introduced to quantify the complexity of care needed by hospitalised patients incorporating factors such as diagnostics chronicity, mental health, social vulnerability and care coordination **[8, 13].**

In contrast, there is an almost universal consensus on the definition of *frailty*, the state of vulnerability, which is intrinsic to the aging process **[14].** Tools for frailty screening are available to clinicians to assess the presumed frail patient on hospital admission **[15, 16].** Very recently, an electronic frailty index (eFI) has been developed to be conveniently obtainable from universally coded health care records **[15].**

It is recognised that the state of frailty and complexity are not interchangeable concepts, and they can coexists as can both exist one without the other **[17].** While there are processes in place to identify the frail patient, a gap exists in the identification of the non-frail but complex hospitalised elderly patient, often presenting high levels of multi-morbidity.

While multi-morbidity in the elderly is highly prevalent, estimates of its prevalence within the hospital setting are scarce and more so among the elderly **[18–21].** Yet, it is well known that multi-morbidity among the elderly is associated with poor hospital outcomes: decreased quality of life, psychological distress, longer hospital stays, more postoperative complications, higher cost of care and higher mortality **[22].**

As part of a comprehensive assessment at point of admission, an elderly patient may benefit from a flagging system which identifies a critical level of multi-morbidity to deliver a more informed care and discharge plan; it is not known, however, at what level of multi-morbidity an elderly patient is at risk of reaching *complexity*.

Thus, our objectives are to take advantage of the large number of hospital administrative records in England in order to:

1. Empirically identify the complex elderly group within the hospital setting based on multi-morbidity and disease burden indicators
2. Examine whether the so-identified complex elderly show evidence of having significantly poorer outcomes (readmissions, length of stay, mortality rates) compared with the rest of the hospital elderly admitted patients.
3. Compare a previously used definition of the complex elderly, with our own empirical identification

## Methods

We used routinely collected hospital administrative data in England, the Hospital Episode Statistics (HES) dataset. We extracted records for patients admitted in the financial year 2012/13. Each record in the dataset represents an episode, i.e., a continuous period of care under one physician. The episodes are linked to *spells* (continuous period of care in one hospital) and to *superspells* (spells in other hospitals due to transfers). We extracted finished inpatient episodes for patients aged at least 65 years and excluded transfers to/from other hospitals. All patient records were anonymized prior to analysis.

We defined multi-morbidity as the simultaneous presence of at least 2 conditions from a list of 20 major conditions including chronic illnesses (**Table A in S1 File**). This list comprised conditions with a prevalence of at least 1% and defined in the Charlson and Elixhauser sets of comorbidity groups **[23–25],** which assigns ICD-10 clinical diagnoses codes to each medical condition. For descriptive statistics, categorical variables (i.e. gender, age groups, diagnoses codes, deaths, and readmissions) were described in terms of counts/frequencies; for quantitative variables (Charlson comorbidity score, length of stay), means and standard deviations were calculated. For reporting outcome measures, we counted emergency admissions and readmissions within 28 days and calculated mean length of stay and in-hospital 30-day mortality rates. A *p* value of less than 0.05 was considered to be significant.

Correspondence Analysis (CA) was used to identify associations between multi-morbidity and medical conditions. See **Section A in S1 File** for details on this Methodology.

For further analysis, the HRG grouping was also dichotomised (present or absent) to indicate whether a patient (record) is considered to be a *complex elderly* under the HRG definition. We calculated the burden of disease, or co-morbidity burden, by taking each targeted condition and calculating the proportion of patients having the condition at each level of multi-morbidity.

We used SAS software version 9.2 to carry out all statistical analysis. We used the PROC CORRESP procedure to carry out the correspondence analysis.

## Results

**Fig 1:**
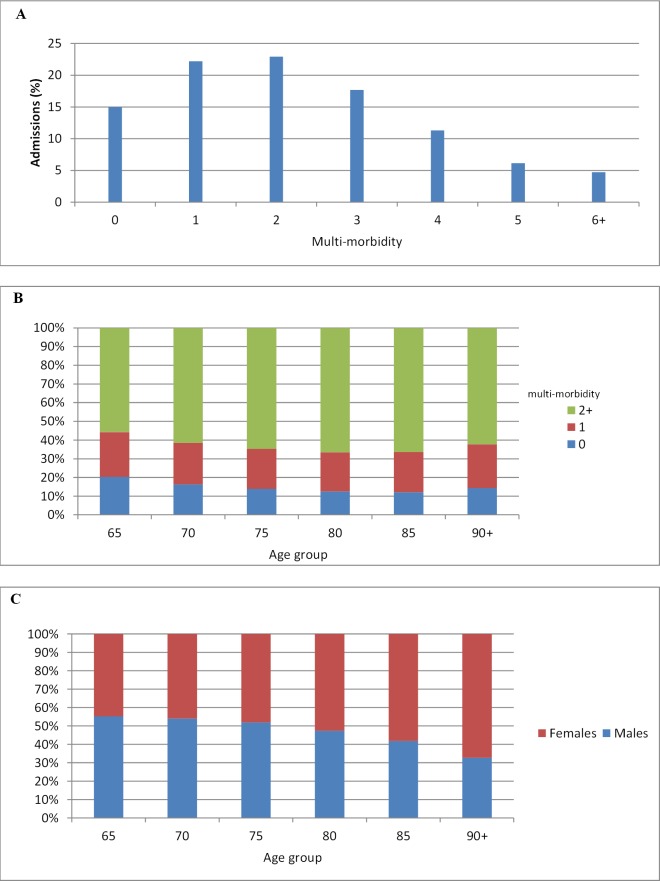
A) Distribution of multi-morbidity in hospitalised elderly patients B) Multi-morbidity by age group C) Gender prevalence by age for patients with multi-morbidity of 2+.

Our sample consisted of a total of 2788900 hospital admissions. 52.8% were females (mean age±SD of 77.5±8.3 years) and the rest were males (mean age±SD of 76.0±7.5 years) (**Table 1**). 62.8% had 2 or more conditions from our list, while 4.7% had 6 or more (**Table 1 and Fig 1A**). The overall average morbidity (at least 1 medical condition from target list) in our sample was 2.7±1.5, and the average multi-morbidity (at least 2 coexisting medical conditions) was 3.2±1.2.

**Table 1:** Sample characteristics.

Characteristic Total (%)	Total 2 788 900 (100)	Male1 315 518 (47.2)	Female 1 473 382 (52.8)
**Age Group (years)**	**Number of admissions (%)**		
**(65–69)**	505 580 (18.2)	271 016 (53.6)	234 564 (46.4)
**(70–74)**	497 684 (17.8)	261 535 (52.6)	236 149 (47.4)
**(75–79)**	539 742 (19.4)	273 924 (50.8)	265 818 (49.2)
**(80–84)**	532 789 (19.1)	246 145 (46.2)	286 644 (53.8)
**(85–89)**	421 689 (15.1)	171 197 (40.6)	250 492 (59.4)
**90 and over**	291 416 (10.4)	91 701 (31.5)	199 715 (68.5)
Comorbidity score*			
**0**	1 111 077 (39.8)	486 783 (43.8)	624 294 (56.2)
**1–3**	193 938 (7.0)	98 618 (50.9)	95 320 (49.1)
**4–5**	335 919 (12.0)	155 281 (46.2)	180 638 (53.8)
**6 and over**	1 147 966 (41.2)	574 836 (50.1)	573 130 (49.9)
**Multi-morbidity** ^**1**^			
**0**	418 274 (15.0)	188 387 (45.0)	229 887 (55.0)
**1**	619 265 (22.2)	281 797 (45.5)	337 468 (54.5)
**2**	639 626 (22.9)	302 619 (47.3)	337 007 (52.7)
**3**	493 287 (17.7)	237 539 (48.2)	255 748 (51.8)
**4**	315 133 (11.3)	153 801 (48.8)	161 332 (51.2)
**5**	171 623 (6.2)	84 743 (49.4)	86 800 (50.6)
**6+**	131 692 (4.7)	66 632 (50.6)	65 060 (49.4)
**2+**	1 751 361 (62.8)	845 334 (48.3)	906 027 (51.7)
**3+**	1 111 735 (39.9)	542 715 (48.8)	569 020 (51.2)

*comorbidity score using the Charlson index and derived from secondary diagnosis fields and specific to English data.

^1^ Count of comorbidities listed in Table A in S1 File

Multi-morbidity rose up to the age of 80, declining thereafter (**Fig 1B**). The proportion of female to male patients with 2 or more coexisting medical conditions increased steadily with age, with more than two thirds being female patients aged 90 and over (**Fig 1C**).

We found that certain medical conditions were highly prevalent in the elder hospital population; **Fig A in S1 File** shows the prevalence of our list of medical conditions. Over 45% of all admitted older patients had hypertension, about 20% had lung conditions, closely followed by myocardial infarction and diabetes (with complications) at 19%, and 13% had some form of cancer (malignant neoplasm).

**Table** B in S1 File presents outcome measures by age-group and multi-morbidity level. As expected, increase age and level of multi-morbidity were positively correlated with poorer outcomes.

## Correspondence Analysis

**Fig 2:**
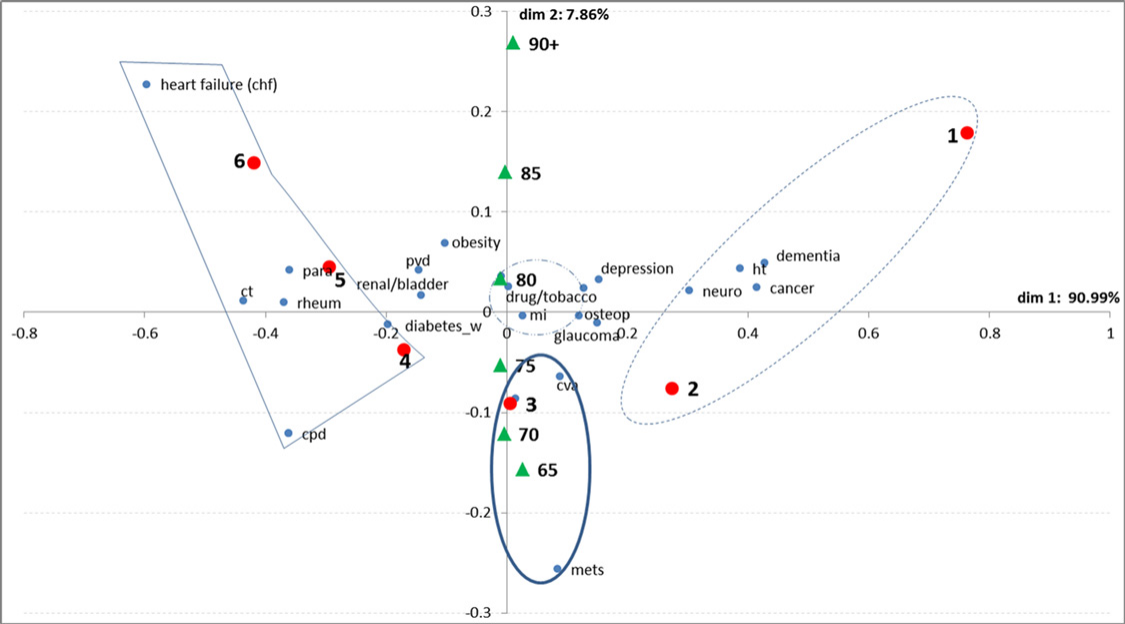
Bi-plot for multi-morbidity level and targeted medical conditions. Group 1 (dashed line); Group 2 (thick line); Group 3 (thin line). Dot-dashed line corresponds to average profiles. Blue dots represent the position of medical conditions on the bi-plot. Red dots represent multi-morbidity levels and green triangles represent the age groups as supplementary variables.

**Fig 2**is a visual representation of the relationship between each medical condition and multi-morbidity with the added information on age group. See **Section B in S1 File** for a detailed explanation of this plot.

We identify the following:

**Group 1.** On the positive side of dimension one; we have older patients with multi-morbidity of 1 and 2 associated with conditions such as dementia, cancer, hypertension and neurological conditions.

**Group 2.** Contributing to dimension 2, we find patients with multi-morbidity of 3 and associated with conditions such as malignant neoplasms, diabetes (without complications) and cerebrovascular disease.

**Group 3.** In opposition, on the negative side of dimension 1, we find patients with higher multi-morbidity levels (4, 5 and 6+) strongly associated with heart failure (including congestive heart failure), lung conditions (incl. asthma and chronic pulmonary diseases), rheumatoid arthritis, and connective tissue disorders (incl. arthritis). Categories located near the centroid of the diagram have average distributions and contribute weakly to either dimension; these include myocardial infarction, gastric ulcers, drug abuse among others (<1%).

Group 1 made up 45.1% of our sample. Over 33% of patients in this group had hypertension recorded, 10% had cancer and 7% with dementia. Group 2 of medium multi-morbidity comprised those having multi-morbidity of 3 and making 17.7% of the sample. In this group, 25% had diabetes (without complications), 10% with cerebrovascular disease (stroke) and 6% with malignant melanoma. Finally, Group 3 with the highest multi-morbidity (4, 5, 6_+_), which is placed in strong contrast to Group 1, makes 22.2% of our sample. Over 64% of patients in this group had heart failure (including congestive heart failure), 54% with lung conditions (incl. asthma and chronic pulmonary diseases), 10% with connective tissue disorders and 11% with rheumatoid arthritis.

**Table C in S1 File** compares outcomes for the multi-morbid groups and for the ‘*healthy*’ elderly, Group 0, those elderly patients in our sample who were admitted to hospital but present none of our list of medical conditions (but may have been diagnosed with other non-major or non-chronic conditions). When comparing these, we observe significant differences (*p*<0.0001) among the four groups. Performing Tukey’s HSD test reveals that Groups 2 and 3 have the smallest significant differences between them but the largest compared with Groups 1 and 0; furthermore, Groups 2 and 3 present the worse outcomes. The proportion of emergency admissions for Groups 2 and 3 is 20% and 26% higher respectively compared with Group 0. The mean length-of-stay is also longer; patients in Group 2 have lengths of stay 45% longer compared with Group 0, increasing to 60% longer for patients in Group 3. Mortality followed a similar pattern. Readmission rates are 1.4 and 1.7 times higher in Groups 2 and 3 respectively compared with Group 0. Thus, based on multi-morbidity level, we identify 2 groups of patients (Groups 2 and 3) within the elderly hospital population who are at greater risk of having adverse outcomes and could be identified as *the complex elderly*.

The association between age and medical conditions shows that the hospitalised elderly patients within the 65–69, 70–74 and 75–79 age groups tend to have medical conditions such malignant melanomas, diabetes (without complications), cerebrovascular diseases and lung conditions (including asthma and chronic pulmonary diseases). The eldest elderly in the age groups 80 and above are more likely to have conditions such as dementia, obesity, depression, heart failure (including congestive heart failure), peripheral vascular disease and neurological diseases.

### Co-morbidity Burden

Co-morbidity burden is defined as the proportion of patients with an index medical condition having 1 or more conditions from our target list. **Table D in S1 File** presents the co-morbidity burden for all listed conditions per number of co-morbidities. Some conditions such as hypertension, cancer, and dementia have a low co-morbidity burden, others such as heart failure (incl. chronic), rheumatoid arthritis, connective tissue disorders present higher burden; a larger number of medical conditions present an intermediate burden.

**Table E in S1 File** presents the main clinical outcomes for the identified complex elderly (Groups 2 and 3) and for the complex elderly identified under the HRG definition used in the past. The HRG group is comprised of a smaller proportion of patients compared to our own. We also include the non-complex elderly from our study, those elderly patients in Groups 0 and 1. We observe significant differences between these 3 groupings.

## Discussion

Our study shows that 22.2% of the elderly hospital population has been diagnosed with one major medical condition, whereas a substantial larger proportion, 62.8%, have 2 or more. Based on the association of multi-morbidity level and medical conditions, we have identified 3 distinct groups of elderly patients in English hospitals. Group 1 is made up of elderly patients with low multi-morbidity and associated with medical conditions having low disease burden; this Group also shows the best outcomes. Group 2 is characterised by patients with medium multi-morbidity and conditions of medium disease burden. Finally, Group 3 is made up of patients with the highest multi-morbidity (3^+^) and having medical conditions with the highest disease burden; this group shows the worse outcomes.

The *complex elderly* label has been used rather loosely in the past and has lacked a consistent definition; thus, to fulfil our first objectives, we propose an empirical prescription for their identification in the hospital setting as those patients within Groups 2 and 3, i.e., *hospitalised patients*, *≥65 years old*, *who have at least 3 simultaneous diagnoses of major clinical conditions*. Second, examination of this Group, shows that the complex elderly thus identified account for 39.9% of our sample, or 1111735 admissions in a single year and show significant (*p*<0.0001) worse clinical outcomes than the non-complex older patient cohort (Groups 0 and 1, **Table E in S1 File**). This proposed definition could be used as part of routinely collected administrative data for hospital resource planning, health service research and risk stratification. Once the definition for the complex elderly in the hospital setting is in place, it could be used to determine and compare outcomes across institutions as an indicator of quality of care. Furthermore, as it is believed that hospital resource utilisation is overall higher for elderly compared with young patients **[26],** it is imperative to clearly identify those complex elderly patients who will potentially require substantial more resources.

**Fig 3:**
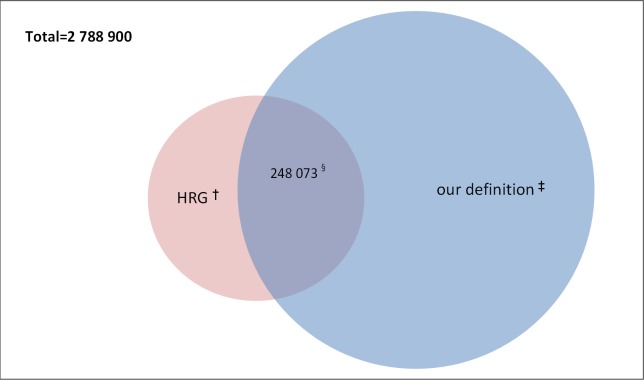
Venn diagram illustrating the overlap between the HRG definition and our proposed definition for complex elderly patients.

With regards to our third objective, we note that outcome indicators for the HRG complex elderly group are significantly different (*p*<0.0001) from those from our proposed definition (**Table E in S1 File**).This is not a concern. The HRG definition was based on a higher age threshold (at least 69 years.) of patients with at least 2 conditions from a specific list of clinical diagnoses and thus it focused on a smaller cohort of high-risk patients. All those patients considered complex elderly under the HRG definition made up a total of 11.6% (323 396) of the total sample. **Fig 3**illustrates the overlap between both definitions for the complex elderly; our definition captures 76.7% of the HRG complex elderly patients. Our complex elderly definition captures a larger elderly population, almost 3.5 times the one defined by HRG, and therefore, the previously used HRG grouping could have significantly underestimated the real cost of caring for the *complex elderly* within the hospital setting.

By using different approaches, a number of studies have focused on the subject of multi- and co-morbidity. However, data sources vary significantly, and it is difficult to make direct comparisons; nevertheless, there are encouraging common results. For example, we find some agreement with a study that uses GP medical records of patients in a small European community **[27];** they find patterns of co-morbidity according to age; older patients (70 years and above) were clustered around diseases such as cardiac arrhythmias, hypertension, diabetes (with and without complications), heart diseases, cerebrovascular disease, renal failure and congestive heart failure. A high disease burden was found for conditions such as heart failure, cerebrovascular disease, and renal failure among others. Another study which focused on the elderly aged 65 years and over used insurance companies record **[28]** to find 3 patterns of multi-morbidity in the elderly (cardiovascular/metabolic, anxiety/depression/somatoform and pain, and neuropsychiatric disorders); these patterns, however, were not related to specific age subgroups.

Using a general population sample of subjects aged 65–95 years, it has been shown that the effect of social support on mortality increases in subjects with the highest comorbidity; moreover, social support is predictive of long-term mortality in the elderly **[29].** Within the hospital setting however, it is not known to what degree social support is predictive of elderly mortality (see Limitations).

### Limitations

One of the limitations of the present study is the use of administrative data sets. Our results depend highly on the recorded clinical diagnoses, and debate exists as to the accuracy of clinical coding within HES **[30];** thus to minimise this effect we have used the latest available HES data (2012–2013), as we know accuracy in coding practice has greatly improved over the last years. Furthermore, hospitals perform limited validation against other databases, and no systematic chart abstraction exists. However, in England, levels of reported accuracy suggest that routinely collected administrative data are sufficiently robust to support their use in research **[30–32].**

Another limitation concerns the selection of medical conditions included in our analysis; at present, there in no standard list of major or chronic diseases, and thus the listed conditions might not be extensive, but we have tried to include all major chronic diseases which are already defined in terms of common comorbidities indices (Charlson and Elixhauser). Refinements could be made regarding the inclusion of other medical conditions. Definitions of Charlson and Elixhauser comorbidities are available for ICD9. An analysis by gender could also prove useful in providing further clues to improve the care of the oldest patients. Due to lack of information regarding social support within hospital administrative data, we are unable to further investigate the relationship between social support, multi-morbidity and long term mortality in hospitalised elderly patients.

## Conclusions

Our study has been based on administrative records from English hospitals but could easily be applied to other healthcare systems where administrative data are routinely gathered. We have identified 3 Groups of older patients who present significant different outcomes and clinical profiles. Based on our findings, we propose an easily replicable approach to identify the *complex elderly* in the acute hospital setting, as those admitted patients, aged 65 years and older with 3 or more co-existing diagnoses from a target list of medical conditions (Groups 2 and 3). These patients present with high disease burden and are commonly diagnosed with heart failure (including chronic), rheumatoid arthritis, connective tissue disorders, pulmonary disease and diabetes.

For the purposes of administrative hospital costs, it would be desirable to re-evaluate and re-introduce a *complex elderly* definition; to this effect we provide a method and justification. Given the trend of increasing admissions of elderly patients to hospitals, there is great demand for a more cost-effective care of hospitalised patients, particularly of those who consume a substantial amount of resources. A revised *complex elderly* definition would not only benefit the patient themselves, but all those involved in their management and care.

### Ethical Approval

We have permission from the NIGB under Section 251 of the NHS Act 2006 (formerly Section 60 approval from the Patient Information Advisory Group) to hold confidential data and analyse them for research purposes. We have approval to use them for research and measuring quality of delivery of healthcare, from the South East Ethics Research Committee.

## Supporting Information

S1 File Table A. Coding definitions (ICD-10 diagnosis codes) for medical conditions in our study. Table B. Outcomes according to age group and multi-morbidity level. Table C. Characteristics and outcomes for identified multi-morbidity Groups. Table D. Co-morbidity burden for all medical conditions targeted in our sample per number of co-morbidities. Table E. Comparison of outcomes between the non-complex elderly^†^, our proposed definition of complex elderly^†^, and the complex elderly as defined by HRG^*^. Fig A. Prevalence of targeted medical conditions in elderly hospital patients. Section A. Correspondence analysis methodology. Section B. Explanation of CA bi-plot.
(DOCX)
