# Supplementary material for: Multi-Morbidity in Hospitalised Older Patients: Who Are the Complex Elderly?
Source: PLoS One. 2015 Dec 30;10(12):e0145372. doi: 10.1371/journal.pone.0145372 (PMC4696783; doi:10.1371/journal.pone.0145372)
Supplement: S1 File — Fig A. Prevalence of targeted medical conditions in elderly hospital patients. Section A. Correspondence analysis methodology. Section B. Explanation of CA bi-plot. (DOCX) [file pone.0145372.s001.docx]

**Table A.** Coding definitions (ICD-10 diagnosis codes) for medical conditions in our study

|  | **ICD-10 diagnosis codes**  **FY 2012**  **Totals (% of adult admissions (> 25) in brackets)**  6 498 665  21 501 680  2 582 167 (39.7)  1 502 239 (23.1)  9 538 848 (44.4)  5 427 681 (25.2)  **Totals, % (percentage of elderly admissions (> 65) in brackets)**  1 193 728 (25.2)  3 970 849 (41.6)  800 564 (16.9)  947 (5.5)  3 041 799 (32.9)  4688 (7.6)  433 738 (9.2)  1088 (5.4)  1 794 571 (18.8)  7721 (5.6)  154 137 (3.3)  731 629 (7.7) |
| --- | --- |
| **Hypertension** | I10, I11, I12, I13, I15 |
| **Renal failure and**  **Bladder disorders** | N03, N052, N053, N054, N055, N056, N072, N073, N074, N01, N18, N25, I12, I13,  I120, I131, N19, N250, Z490, Z491, Z492, Z940, Z992,N32, S37 |
| **Lung (including chronic pulmonary) conditions -asthma** | I278, I279, J40, J41, J42, J43, J44, J45, J46, J47, J60, J61, J62, J63, J64, J65, J66, J67, J684, J701, J703 |
| **Myocardial infarction** | I21, I22, I23, I252, I258 |
| **Diabetes** With complications | E102, E112, E132, E142, E103, E113, E133, E143, E104, E114, E134, E144, E107, E117, E137, E147 |
| **Cancer** | C0,C1,C2,C3,C40,C41,C43,C45,C46,C47,C48,C49,C5,C6,C70,C71,C72,C73,C74,C75,C76,C80,C81,C82,C83,  C84,C85,C883,C887,C889,C900,C901,C91,C92,C93,C940,C941,C942,C943,C945,C947,C95,C96,C880,C881,  C882,C902,C944,C97 |
| **Peripheral vascular disease** | I71, I790, I739, R02, Z958, Z959 |
| **Cerebrovascular disease (stroke)** | I60,I61,I62,I63,I65,I66,G450,G451,G452,G458,G459,G46,I64,G454,I670,I671,I672,I674,I675,I676I677,I678, I679,I681,I682,I688,I69,I673,I680 |
| **Neurological conditions (including Parkinson’s, Epilepsy, MS)** | G10,G11,G12,G13,G21,G22,G254,G255,G312,G318,G319,G32,G35,G36,G37,G40,G41,G931,G934,R470,R56,  G20 |
| **Glaucoma** | H40, H42 |
| **Osteoporosis** | M80, M81, M82, M83, M84, M85 |
| **Depression** | F204,F313,F314,F315,F32,F33,F341,F412,F432 |
| **Obesity** | E66 |
| **Drug abuse (incl. tobacco)** | F11,F12,F13,F14,F15,F16,F18,F19,Z715,Z722,F17 |
| **Dementia and Alzheimer’s** | F00, F01, F02, F051, F03,  G30X, G310, G311 |
| **Heart failure (incl. congestive)** | I099, I110, I130, I132, I255, I420, I425, I426, I427, I428, I429, I43, I50, P290 |
| **Rheumatoid arthritis** | L940,L941,L943,M05,M06,M08,M120,M123,M30,M310,M311,M312,M313,M32,M33,M34,M35,M45,M461, M468,M469 |
| **Connective tissue (incl. arthritis)** | M32,M34,M332,M053,M058,M059,M060,M063,M069,M050,M052,M051,M353 |
| **Paraplegia** | G81,G041,G820,G821,G822 |
| **Malignant neoplasm** | C77,C78,C79,C80 |

**Table B.** Outcomes according to age group and multi-morbidity level

| **Multi-morbidity** | **Age groups**  (Admissions) | | | | | | | | | | |  |  |
| --- | --- | --- | --- | --- | --- | --- | --- | --- | --- | --- | --- | --- | --- |
|  | **65**  (505 580) | | **70**  (497 684) | **75**  (539 742) | | **80**  (532 789) | | **85**  (421 689) | | **90+**  (291 416) | |  |  |
|  |  | |  |  | |  | |  | |  | |  |  |
|  | **Emergency admissions (%)** | | | | | | | | | | |  |  |
| 0 | 57.0 | 59.3 | | | 64.6 | | 74.5 | | 84.0 | | 92.7 |  |  |
| 1 | 59.6 | 62.2 | | | 68.0 | | 77.9 | | 86.9 | | 94.4 |  |  |
| 2 | 65.6 | 69.1 | | | 74.0 | | 82.0 | | 89.4 | | 95.6 |  |  |
| 3 | 69.8 | 73.0 | | | 77.4 | | 84.9 | | 91.0 | | 96.1 |  |  |
| 4 | 73.4 | 77.3 | | | 81.2 | | 87.5 | | 92.8 | | 96.7 |  |  |
| 5 | 76.8 | 80.2 | | | 84.0 | | 89.3 | | 93.8 | | 97.3 |  |  |
| 6+ | 82.0 | 84.8 | | | 87.7 | | 91.9 | | 95.0 | | 97.7 |  |  |
|  | **Mean LOS in days (SD)** | | | | | | | | | | |  |  |
| 0 | 4.3 (18.3) | | 4.9 (30.9) | 5.6 (25.2) | | 6.7 (25.3) | | 7.9 (17.8) | | 8.6 (15.9) | |  |  |
| 1 | 5.2(20.0) | | 5.8 (26.6) | 6.8 (24.7) | | 8.1 (25.2) | | 9.2 (22.6) | | 10.0 (24.4) | |  |  |
| 2 | 5.6 (12.5) | | 6.2 (16.8) | 7.1 (14.7) | | 8.4 (14.1) | | 10.0 (15.0) | | 10.8 (14.6) | |  |  |
| 3 | 6.1 (11.9) | | 6.9 (13.3) | 7.9 (15.8) | | 9.3 (15.0) | | 10.7 (15.4) | | 11.7 (15.6) | |  |  |
| 4 | 6.9 (12.1) | | 7.7 (16.2) | 8.7 (13.9) | | 10.0 (14.3) | | 11.3 (15.2) | | 12.1 (15.3) | |  |  |
| 5 | 7.6 (17.4) | | 8.3 (13.1) | 9.5 (13.7) | | 10.7 (14.7) | | 12.0 (15.3) | | 12.5 (14.9) | |  |  |
| 6+ | 8.5 (12.3) | | 9.3 (13.3) | 10.4 (17.9) | | 11.5 (14.8) | | 12.4 (15.3) | | 13.0 (15.4) | |  |  |
|  | **30 day in-hospital mortality rate (%)** | | | | | | | | | | |  |  |
| 0 | 0.6 | | 0.9 | 1.3 | | 2.3 | | 3.6 | | 6.0 | |  |  |
| 1 | 1.5 | | 1.9 | 2.5 | | 3.6 | | 5.4 | | 8.5 | |  |  |
| 2 | 2.8 | | 3.3 | 4.0 | | 5.5 | | 7.5 | | 11.1 | |  |  |
| 3 | 3.5 | | 4.3 | 5.3 | | 7.1 | | 9.5 | | 13.6 | |  |  |
| 4 | 4.6 | | 5.4 | 7.1 | | 9.1 | | 11.8 | | 16.0 | |  |  |
| 5 | 5.9 | | 7.2 | 8.6 | | 11.0 | | 13.5 | | 17.7 | |  |  |
| 6+ | 8.0 | | 9.5 | 10.8 | | 13.6 | | 16.2 | | 19.0 | |  |  |
|  | **28-day readmission rate (%)** | | | | | | | | | | |  |  |
| 0 | 7.9 | | 8.9 | 10.2 | | 12.3 | | 14.8 | | 16.1 | |  |  |
| 1 | 9.8 | | 10.2 | 11.1 | | 12.7 | | 14.6 | | 15.7 | |  |  |
| 2 | 12.1 | | 12.7 | 13.5 | | 14.7 | | 15.8 | | 16.4 | |  |  |
| 3 | 14.0 | | 14.5 | 15.0 | | 16.0 | | 16.9 | | 16.8 | |  |  |
| 4 | 15.4 | | 16.3 | 17.0 | | 17.5 | | 18.1 | | 17.7 | |  |  |
| 5 | 17.3 | | 17.7 | 18.6 | | 18.6 | | 19.3 | | 18.4 | |  |  |
| 6+ | 20.7 | | 20.8 | 21.1 | | 21.1 | | 20.7 | | 19.5 | |  |  |

**Table C.** Characteristics and outcomes for identified multi-morbidity Groups

|  | **Group 0**  **Multi-morbidity=0** | **Group 1**  **Multi-morbidity=1,2** | **Group 2**  **Multi-morbidity=3** | **Group 3**  **Multi-morbidity=4,5,6** |
| --- | --- | --- | --- | --- |
| **Mean age (years)± SD** | 77.2± 8.7 | 78.4±8.4 | 78.7±8.1 | 79.0±8.4 |
| **Admissions (%)** | 418 274 (15.0) | 1 258 891 (45.1) | 493 287 (17.7) | 618 448 (22.2) |
| **Emergencies (%)** | 286 189 (68.0) | 948 983 (75.4) | 399 621 (81.0) | 531 548 (85.9) |
| **LOS (days) ± SD** | 5.9±23.4 | 7.5±19.9 | 8.6±14.7 | 9.8±14.8 |
| **30-day in-hospital death (%)** | 8 184 (1.9) | 54 986 (4.4) | 33 041 (6.7) | 60 883 (9.8) |
| **28-day readmissions (%)** | 45 430 (10.9) | 164 019 (13.0) | 76 220 (15.5) | 112 433 (18.2) |
| **Comorbidity score± SD** | 0.0±0.3 | 4.3±5.7 | 9.2±7.1 | 16.3±9.7 |

**Table D.** Co-morbidity burden for all medical conditions targeted in our sample per number of co-morbidities

| **Medical condition** | **Total cases** | **condition +1 more**  **(%)** | **condition +2 more**  **(%)** | **condition +3 more**  **(%)** | **condition +4 more**  **(%)** | **condition +5 or more**  **(%)** |
| --- | --- | --- | --- | --- | --- | --- |
| **Hypertension**  **Cancer**  **Neurological conditions**  **Dementia-Alzheimer’s**  **Malignant neoplasms**  **Glaucoma**  **Osteoporosis**  **Cerebrovascular diseases**  **Drug and tobacco abuse**  **Myocardial infarction**  **Diabetes (with*)**  **Renal and bladder disorders**  **Peripheral vascular disease**  **Obesity**  **Paraplegia**  **Heart failure (incl. chronic)**  **Chronic pulmonary disease**  **Rheumatoid arthritis**  **Connective tissue disorders** | 1 272 599  372 247  169 045  267 594  123 881  37 095  121 793  207 023  171 552  519 349  25 067  333 423  117 733  53 331  33 598  267 835  569 505  106 634  96 047 | 25.2  26.9  24.6  26.1  30.7  23.1  21.9  23.5  17.4  21.3  16.9  18.0  17.2  18.6  12.5  7.7  14.8  14.7  13.7 | 22.8  22.5  23.0  22.6  25.5  23.3  23.4  24.3  24.1  22.8  24.8  22.7  22.7  21.7  23.2  17.6  26.4  22.9  22.8 | 15.9  15.5  16.7  15.2  18.6  18.1  18.8  18.8  20.5  19.4  20.5  20.7  20.5  19.5  23.5  23.9  25.1  23.0  23.5 | 9.2  8.9  10.0  8.7  10.7  11.8  12.1  11.9  13.9  13.2  15.5  15.2  15.1  14.0  17.7  22.9  17.1  17.4  18.1 | 7.7  7.1  7.9  7.2  8.9  10.9  11.3  11.8  13.1  13.2  17.1  16.6  17.3  17.9  20.3  28.5  16.6  20.7  22.0 |

* Diabetes is divided into with and without long-term complications

**Table E.** Comparison of outcomes between the non-complex elderly^†^, our proposed definition of complex elderly^†^, and the complex elderly as defined by HRG^*^

| **Total sample = 2 788 900** | | |  |
| --- | --- | --- | --- |
|  | **Non-complex elderly† – this study**  **(1 667 165, 60.1%)** | **Complex Elderly^‡^**  **– this study**  **(1 111 735, 39.9%)** | **HRG^*^**  **Complex elderly**  **(323 396, 11.6%)** |
| **Mean age (years) ± SD** | 77.8±6.1 | 78.9±8.0 | 83.0±6.8 |
| **Emergencies (%)** | 1 235 172 (74.1) | 931 169 (83.8) | 319 999 (98.9) |
| **LOS (days) ± SD** | 6.7±15.4 | 9.3±14.8 | 13.4±17.7 |
| **30-day in-hospital deaths (%)** | 63 170 (3.8) | 93 924 (8.4) | 57 820 (17.9) |
| **28-day readmissions (%)** | 209 449 (12.6) | 188 653 (17.0) | 57 433 (17.8) |
| **Comorbidity score ± SD** | 2.2±2.9 | 13.1±9.3 | 16.3±10.4 |

† Non-complex elderly corresponds to Groups 0 and 1 in our study

‡ Complex elderly patients identified by our analysis correspond to Groups 2 and 3

* HRG: Healthcare Resource Group, v3.5

**Fig A.** Prevalence of targeted medical conditions in elderly hospital patients

*Diabetes-without: diabetes without complications

Section A

Correspondence Analysis is a non-parametric multivariate exploratory technique for categorical data analysis **[1-4].** This technique permits a graphical representation of the rows and columns in a cross tabulation of variables (contingency table). Each row and column is depicted as a single point on a scatter plot (bi-plot). The positions on the bi-plot, commonly referred to as map, reveal associations or similarities between variable categories through their proximity. For example, opposite points on the map indicate dissimilarity between categories.

This method required the following steps:

- The 20 medical conditions were dichotomised, thus creating a separate variable for each of them. Thus, a total of 20 variables representing the major medical conditions in our analysis were created.
- Multi-morbidity categorization: this new variable was categorised from 0 (patient having none of the major medical conditions in our list), up to 6 (patient having 6 or more simultaneously).
- The age group variable was categorised in 6 groups: 65, 70, 75, 80, 85 and 90+

Thus, a total of 22 variables (20 medical conditions, multi-morbidity and age group) were used in correspondence analysis.

The information contained in the CA cross-tabulation can be fully represented in a multi-dimensional space, which can be reduced to the more manageable 2 or 3 dimensions, due to the association between rows and columns. We created a frequency table where rows represent multi-morbidity level from 1 up to 6 (6+), and columns represent the 20 targeted medical conditions. Supplementary rows corresponding to each age group category were also included to aid interpretation of the result. Because categories having equal profiles (distribution along row or column) are placed at the centroid of the plot, we excluded the category corresponding to multi-morbidity of zero, which represents all those elderly patients in our sample having NONE of the targeted medical conditions (this row would be made up of the same number for each medical condition).

The procedure then calculates several statistical parameters to evaluate and characterise each dimension (absolute and relative contributions to inertia, quality of representation and position on the axis of the dimension). The amount of information explained by each dimension is evaluated using the Benzecri inertia adjustment and Greenacre approximation explained elsewhere **[5,6].**

References

1. Greenacre M, Hastie T. The geometrical interpretation of Correspondence Analysis. **J Am Stat Assoc. 1987**;82(398):437-447
2. Greenacre M. (2007) Correspondence Analysis in Practice. 2^nd^ edition Chapman & Hall/CRC
3. Storti, D. (2010). Correspondence Analysis, from UNESCO. Available at <http://www.unesco.org/webworld/idams/advguide/Chapt6_5.htm>. Accessed March 3, 2015
4. Doey L, Kurta J. Correspondence Analysis applied to Psychological Research. **Tutor Quant Methods Psychol. 2011**;7(1):5-14
5. Benzécri, JP. Sur le Calcul des taux d’inertie dans l’analyse d’un questionnaire. **Cahiers de l’Analyse des Données.** **1979**;4,377-378
6. Greenacre, MJ. (1984), Theory and Applications of Correspondence Analysis, London: Academic Press.

Section B

The plot in **Fig. 2** offers a concise view of the intricate inter-relationship between degree of multi-morbidity and medical conditions; it is dominated by dimension 1 which explains 90.9% of the information and dimension 2, which explains 7.9%. Dimension 3 explains only 1.2% and thus it is excluded from the analysis. Dimension 1 has naturally ordered the multi-morbidity variable in increasing order, from right to left. The supplementary rows for age groups are aligned along dimension 2. By inspecting the relative contributions of rows and columns we are able to interpret the associations between these variables. Because there are 6 row categories (multi-morbidity), any contribution larger than 16.7% would be significantly greater than the expected from a random contribution of categories to the dimension. Similarly for the columns, any contribution larger than 4.2% is considered to be significant. Thus by inspecting inertia contributions for rows and columns we find 3 main levels of contributions to dimensions 1 and 2. Group 1 corresponds to those variables contributing strongly to dimension 1 (contribution is > 32%), Group 2 contributes moderately to dimension 1 and 2 (19% and 22% respectively) and Group 3 which also contributes strongly to dimension 1 (>20%), but opposed to Group 1.
